# The effect of robot-assisted gait training for patients with spinal cord injury: a systematic review and meta-analysis

**DOI:** 10.3389/fnins.2023.1252651

**Published:** 2023-08-22

**Authors:** Luo Bin, Xiaoping Wang, Hu Jiatong, Fan Donghua, Wang Qiang, Shen Yingchao, Miao Yiming, Ma Yong

**Affiliations:** ^1^Department of Orthopaedics, Changshu Hospital Affiliated to Nanjing University of Chinese Medicine, Changshu, China; ^2^Department of Orthopedics, PLA Strategic Support Force Characteristic Medical Center, Beijing, China; ^3^Department of Acupuncture and Moxibustion, Guang'an Men Hospital, China Academy of Chinese Medical Sciences, Beijing, China; ^4^Institute of Traumatology & Orthopedics and Laboratory of New Techniques of Restoration & Reconstruction of Orthopedics and Traumatology, Nanjing University of Chinese Medicine, Nanjing, China; ^5^Department of Traumatology & Orthopedics, Affiliated Hospital of Nanjing University of Chinese Medicine, Nanjing, China

**Keywords:** robot-assisted, gait training, spinal cord injury, gait distance, gait speed

## Abstract

**Background:**

With the aging of the global population, Spinal injuries are often prone to occur and affect human health. The development of technology has put robots on the stage to assist in the treatment of spinal injuries.

**Methods:**

A comprehensive literature search were carried out in multiple databases, including PubMed, Medline (Ovid), Web of Science, Cochrane, Embase, Scopus, CKNI, Wang fang, VIP database, Sino Med, Clinical Trails until 20th, June, 2023 to collect effect of robot-assisted gait training for patients with spinal cord injury patients. Primary outcome includes any changes of gait distance and gait speed. Secondary outcomes include any changes in functions (Such as TUG, Leg strength, 10 MWT) and any advent events. Data were extracted from two independent individuals and Cochrane Risk of Bias tool version 2.0 was assessed for the included studies. Systematic review and meta-analysis were performed by RevMan 5.3 software.

**Results:**

11 studies were included in meta-analysis. The result showed that gait distance [WMD = 16.05, 95% CI (−15.73, 47.83), I^2^ = 69%], gait speed (RAGT vs. regular treatment) [WMD = 0.01, 95% CI (−0.04, 0.05), I^2^ = 43%], gait speed (RAGT vs. no intervention) [WMD = 0.07, 95% CI (0.01, 0.12), I^2^ = 0%], leg strength [WMD = 0.59, 95% CI (−1.22, 2.40), I^2^ = 29%], TUG [WMD = 9.25, 95% CI (2.76, 15.73), I^2^ = 74%], 10 MWT [WMD = 0.01, 95% CI (−0.15, 0.16), I^2^ = 0%], and 6 MWT [WMD = 1.79, 95% CI (−21.32, 24.90), I^2^ = 0%].

**Conclusion:**

Robot-assisted gait training seems to be helpful for patients with spinal cord to improve TUG. It may not affect gait distance, gait speed, leg strength, 10 MWT, and 6 MWT.

## 1. Introduction

### 1.1. Background

Spinal cord injury is a serious neurological injury, the annual global incidence is estimated to be between 250,000 and 500,000 cases (Mekki et al., 2018), usually caused by car accidents, falls, sports accidents, or other external factors. This type of injury can lead to lower limb paralysis or loss of function, which has a significant impact on the quality of life and independence of patients. Previously, spinal cord injury meant being confined to a wheelchair and accompanied by lifelong medical complications. Doctors have very limited treatment options (McDonald and Sadowsky, 2002). Nevertheless, as technology continues to advance, individuals with incomplete para−/tetraplegia can regain the ability (neuroplasticity) to relearn crucial daily activities and reintegrate into the workforce (Dietz and Fouad, 2014). Neuroplasticity refers to the ability of the nervous system to reorganize and adapt in response to changes in demands and environments. This phenomenon occurs during skill acquisition, following nervous system damage, and as a result of sensory deprivation (Bavelier and Neville, 2002; Hötting and Röder, 2013). Neuroplasticity can be promoted through exercise training with the help of the need and/or through electrical stimulation techniques (Alashram et al., 2021). This kind of training helps the existence of physiological Proprioception input of the spinal cord, and the addition of rehabilitation technology (Dietz and Fouad, 2014).

The utilization of neuro-rehabilitation robots in rehabilitation has demonstrated promising clinical outcomes (Hu et al., 2015; Qian et al., 2017; Rong et al., 2017; Demofonti et al., 2021; Selph et al., 2021). Robot-assisted gait training has been shown to enhance neuroplasticity, whereby injured nerves gradually regain their functionality through repetitive training and stimulation (Kuwahara et al., 2022). Secondly, this type of training can enhance the patient’s muscle strength and stability, improve the efficiency and safety of walking (Mıdık et al., 2020). Furthermore, robot-assisted gait training can offer patients confidence and motivation during the initial stages of recovery, thereby reducing feelings of anxiety and depression (Yang et al., 2022). Robot assisted gait training refers to the use of advanced robot technology, combined with the principles of physical therapy, to provide gait training and rehabilitation treatment for patients with spinal cord injury through robot equipment (Calabrò et al., 2021). This training method can provide accurate control and support for patients, helping them recover walking function (Picelli et al., 2021). For example, wearable Exoskeleton or robot walker can provide additional support and stability to assist patients in walking (Swinnen et al., 2010) These devices are usually equipped with sensors and motors that can be adjusted according to the needs and abilities of patients. Through repeated training and gradually increasing challenges, patients can gradually improve muscle strength, balance, and walking coordination (Stampacchia et al., 2022).

However, the effect of robot-assisted gait training for patients with spinal cord injury is still unclear. Therefore, we conduct a systematic review and meta-analysis to assess the effect and safety of robot-assisted gait training for patients with spinal cord injury patients.

## 2. Methods

### 2.1. Database selection and search strategy

Literature searches were conducted in the database of PubMed, Medline (Ovid), Web of Science, Cochrane, Embase, Scopus, CKNI, Wang fang, VIP database, Sino Med, Clinical Trails until 20th, June, 2023. The search strategy of Medline (Ovid) is shown as following (Table 1).

**Table 1:** The search strategy of Medline (Ovid).

Search order	Search strategy
#1	Exp spinal cord injuries/
#2	Exp spinal cord ischemia/
#3	Exp central cord syndrome/
#4	[myelopathy adj3 (traumatic or post-traumatic)].ab,ti.
#5	[(spine or spinal) adj3 (fracture$ or wound$ or trauma$ or injur$ or damag$)].ab,ti.
#6	[spinal cord adj3 (contusion or laceration or transaction or trauma or ischemia)].ab,ti.
#7	Central cord injury syndrome.ab,ti.
#8	Central spinal cord syndrome.ab,ti.
#9	Exp cervical vertebrae/in (Injuries)
#10	Exp spinal cord/
#11	SCI.ab,ti.
#12	Exp paraplegia/
#13	Exp quadriplegia/
#14	(paraplegia* or quadriplegia* or tetraplegia*).ab,ti.
#15	or/1–14
#16	Exp robot-assisted/
#17	Exp robot/
#18	Exp robot-assisted gait training/
#19	Or/16–18
#20	15 and 19

### 2.2. Inclusion criteria

- Randomized controlled trials (RCTs);
- Inclusion of people with spinal cord injury; and
- Patients using robot-assisted gait training as the main treatment or robot-assisted gait training with regular treatment comparing with regular treatment is also acceptable.

### 2.3. Exclusion criteria

- There is no single variable;
- observational studies; and
- lack of sufficient information on baseline

### 2.4. Primary outcome

Any changes of gait distance and gait speed.

### 2.5. Secondary outcomes

Any changes in functions (Such as TUG, Leg strength, 10 MWT).

Any advent events.

### 2.6. Method of data extraction

Two independent reviewers (LB and XB) extracted data using a standardized form that included study demographics, baseline characteristics, study design, intervention methods, outcome measures, and results. Any disagreements were resolved through discussion, and a third review author was consulted if necessary (Sally Green, 2011).

### 2.7. Bias risk assessment

Two authors evaluated the risk of bias in the included study using the Cochrane Handbook for Systematic Reviews of Interventions Version 6.0 (updated July 2019) risk of bias assessment tool. Any discrepancies were resolved through consensus. The assessment tool evaluated seven items, including random sequence generation, assignment concealment, blinding of participants and personnel, blinding of outcome assessment, incomplete outcome data, selective reporting, and other bias. The items were categorized as green, yellow, and red colors and “+,” “−,” “?,” indicating “low,” “high,” and “unclear” risk of bias.

### 2.8. Publication bias assessment

The RevMan 5.3 software was utilized to conduct funnel plots for the assessment of publication bias pertaining to the primary outcome measures.

### 2.9. Statistical analysis

The Review Manager software (RevMan version 5.3, Cochrane Collaboration, Oxford, UK) was utilized to conduct statistical analyses. The effect quantity used to combine continuous variables in the study was Weighted Mean Difference (WMD) and 95% CI.

### 2.10. Heterogeneity analysis

Heterogeneity between trial results was tested using *p* value and I^2^ statistic. In cases where more than two articles were included, heterogeneity was assessed. If the I^2^ > 50%, the random effect model was applied based on Clinical heterogeneity. To evaluate the source of heterogeneity, subgroup, sensitivity analysis, and funnel chart were employed. The statistical calculation process was carried out using RevMan5.3 software.

## 3. Results

### 3.1. Literature search

**Figure 1:**
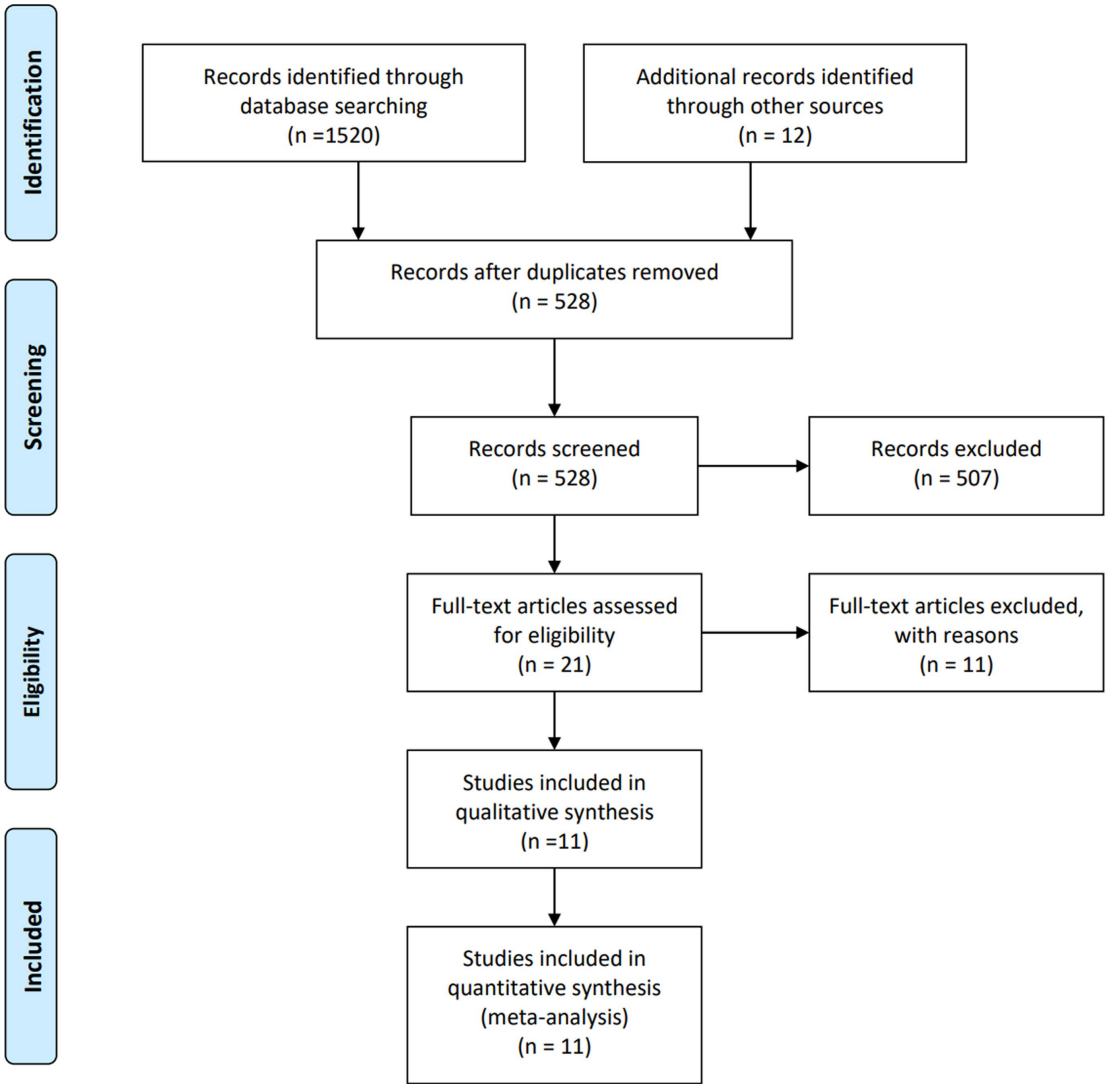
Flowchart of study selection.

We searched 8 databases including PubMed, Medline (Ovid), Web of Science, Cochrane, Embase, Scopus, CKNI, Wang fang, VIP database, Sino Med and Clinical Trails until 20th, June, 2023. 1,520 Records identified through database searching and 12 records identified through other sources. 528 records are collected after duplicates removed. 22 articles are assessed for eligibility and 11studies are finally involved in meta-analysis (Figure 1).

### 3.2. Characteristics of include studies

11 studies characteristics information are collected in Table 2. The difference are discussed by the third author or the whole group.

**Table 2:** Characteristics of included studies.

Study	Participants (*N*)		Intervention		Study design	Outcome measures	Level of injury
	Exp.	Con.	Exp.	Con.			
Hornby et al. (2005)	10	20	RAGT + Usual PT	Usual PT	RCT	leg strength	ASIA B, C, D Level of injury: above T10
Field-Fote and Roach (2011)	14	50	RAGT + Usual PT	Usual PT	RCT	Gait speed; gait distance; leg strength	ASIA C, D Level of injury: At or above T10
Alcobendas-Maestro et al. (2012)	37	38	RAGT + Usual PT	Usual PT	RCT	Gait speed; gait distance; leg strength	ASIA C, D Level of injury: C2 to T12
Esclarín-Ruz et al. (2014)	41	42	RAGT + Usual PT	Usual PT	RCT	Gait speed; gait distance; leg strength	ASIA C, D Level of injury: C2 to L3
Labruyere and van Hedel (2014)	5	4	RAGT + Usual PT	Usual PT	RCT	Gait speed leg; strength	ASIA C, D Level of injury: C4 to T11
Niu et al. (2014)	20	20	RAGT	No intervention	RCT	Gait speed; TUG	ASIA B, C, D Level of injury: above T10
Shin et al. (2014)	27	26	RAGT + Usual PT	Usual PT	RCT	Leg strength	ASIA D Level of injury: UMN
Tang et al. (2014)	15	15	RAGT + Usual PT	Usual PT	RCT	Gait speed	ASIA D Level of injury: T8 to L3
Varoqui et al. (2014)	15	15	RAGT	No intervention	RCT	Gait speed; TUG; 10MWT; 6WT	ASIA C, D Level of injury: above T10
Lam et al. (2015)	8	5	RAGT + Usual PT	Usual PT	RCT	10MWT; 6WT	ASIA C, D Level of injury: lesion level below T11 or lower motoneuron injury was excluded
Duffell et al. (2015)	27	29	RAGT	No intervention	RCT	Gait speed; gait distance; TUG	ASIA C, D Level of injury: above T10

### 3.3. Risk of bias

**Figure 2:**
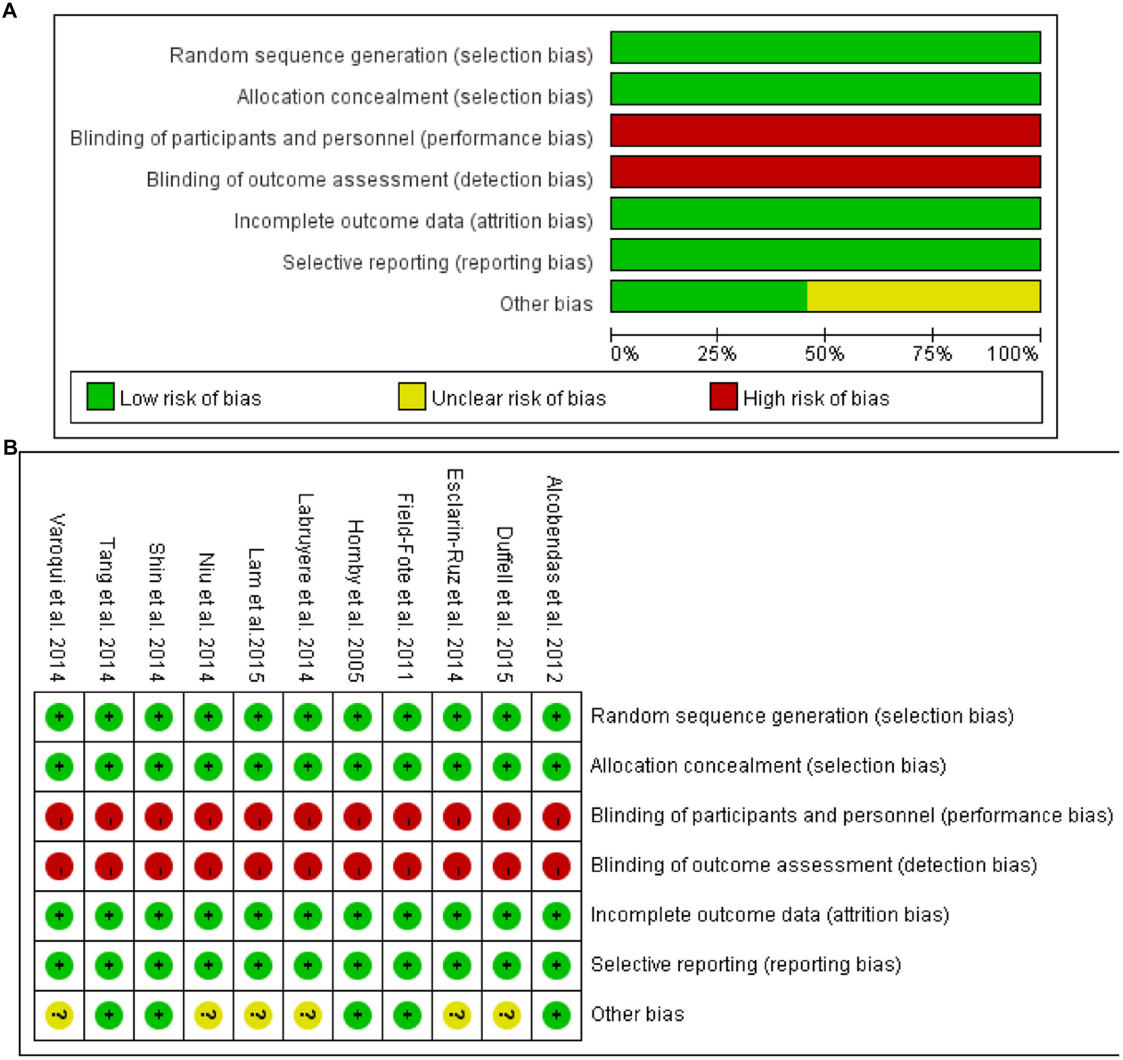
**(A)** Risk of bias graph; **(B)** Risk of bias summary.

All inclueded are low risk of selection bias. Due to using different rehabilitation methods, all the studies are high of performance bias and detection bias. All studies are low risk of attritions bias and reporting bias. Some studies are unclear of other bias such are lost of follow-up (See Figure 2).

### 3.4. Gait distance

**Figure 3:**
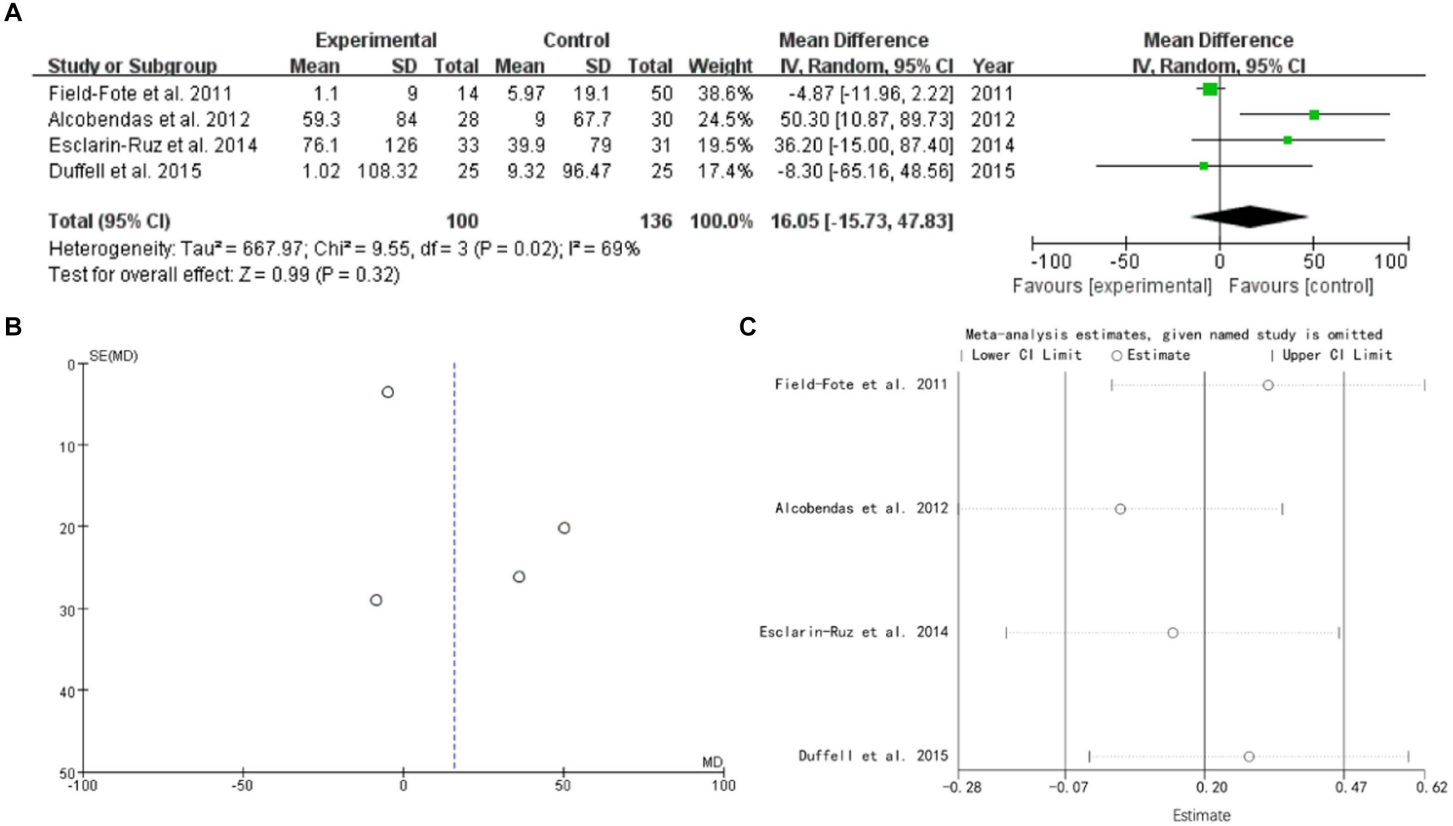
gait distance **(A)** forest plot, **(B)** funnel plot, and **(C)** sensitivity analysis.

4 studies mentioned gait distance. The forest plot weight mean difference WMD = 16.05, 95% CI (−15.73, 47.83), I^2^ = 69%. The funnel plot shows that asymmetric. It may have publication bias. Sensitivity analysis is conducted and show that values included in the literature are all within a reasonable range (Figure 3).

### 3.5. Gait speed

**Figure 4:**
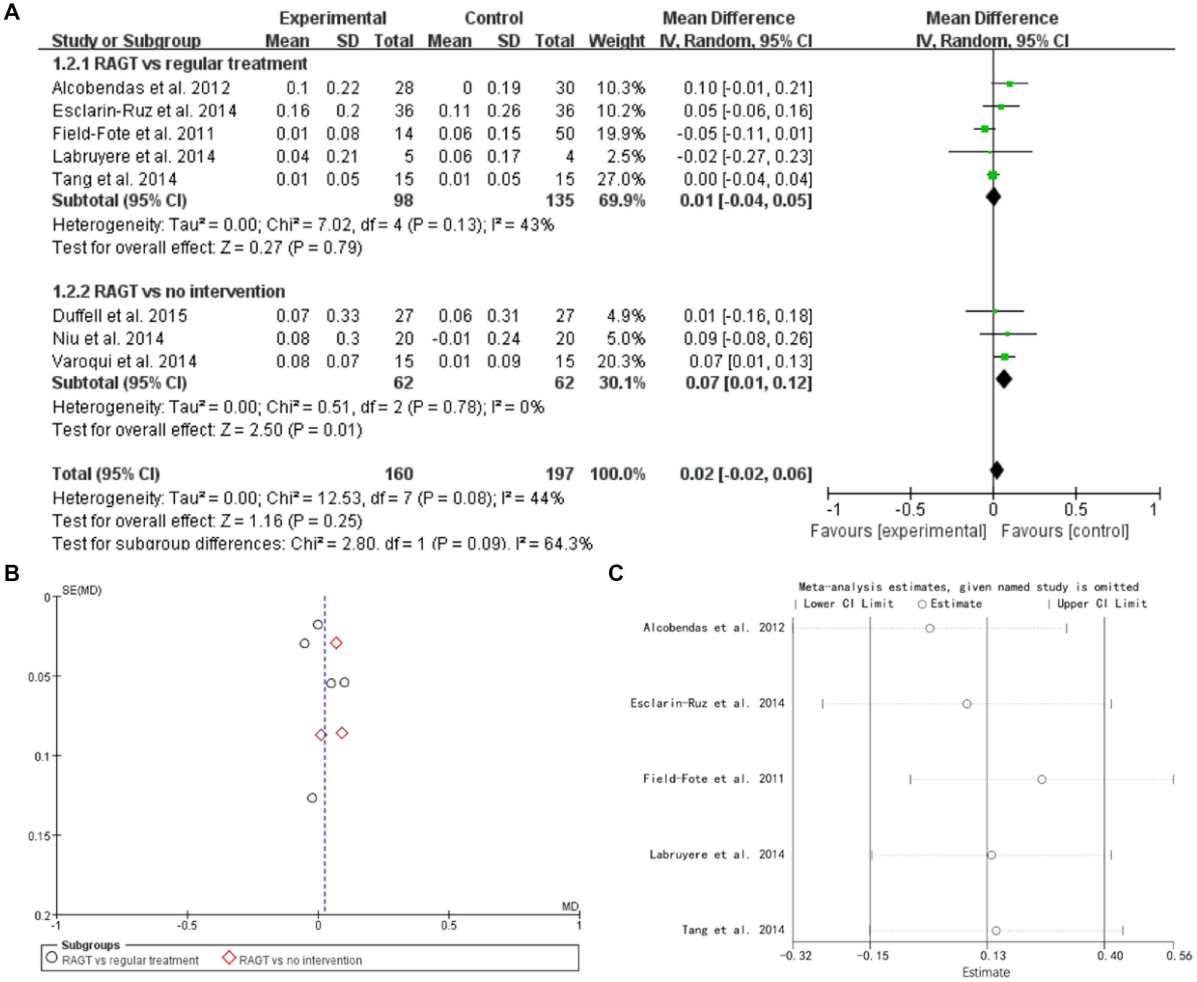
Gait speed **(A)** forest plot, **(B)** funnel plot, and **(C)** sensitivity analysis.

8 studies mentioned gait speed. Subgroud group analysis are used to distinguish different variables. The forest plot weight mean difference (RAGT vs. regular treatment) WMD = 0.01, 95% CI (−0.04, 0.05), I^2^ = 43%. The forest plot weight mean difference (RAGT vs. no intervention) WMD = 0.07, 95% CI (0.01, 0.12), I^2^ = 0%. The funnel plot shows that asymmetric. It may have publication bias. Sensitivity analysis is conducted and show that values included in the literature are all within a reasonable range (Figure 4).

### 3.6. Leg strength

**Figure 5:**
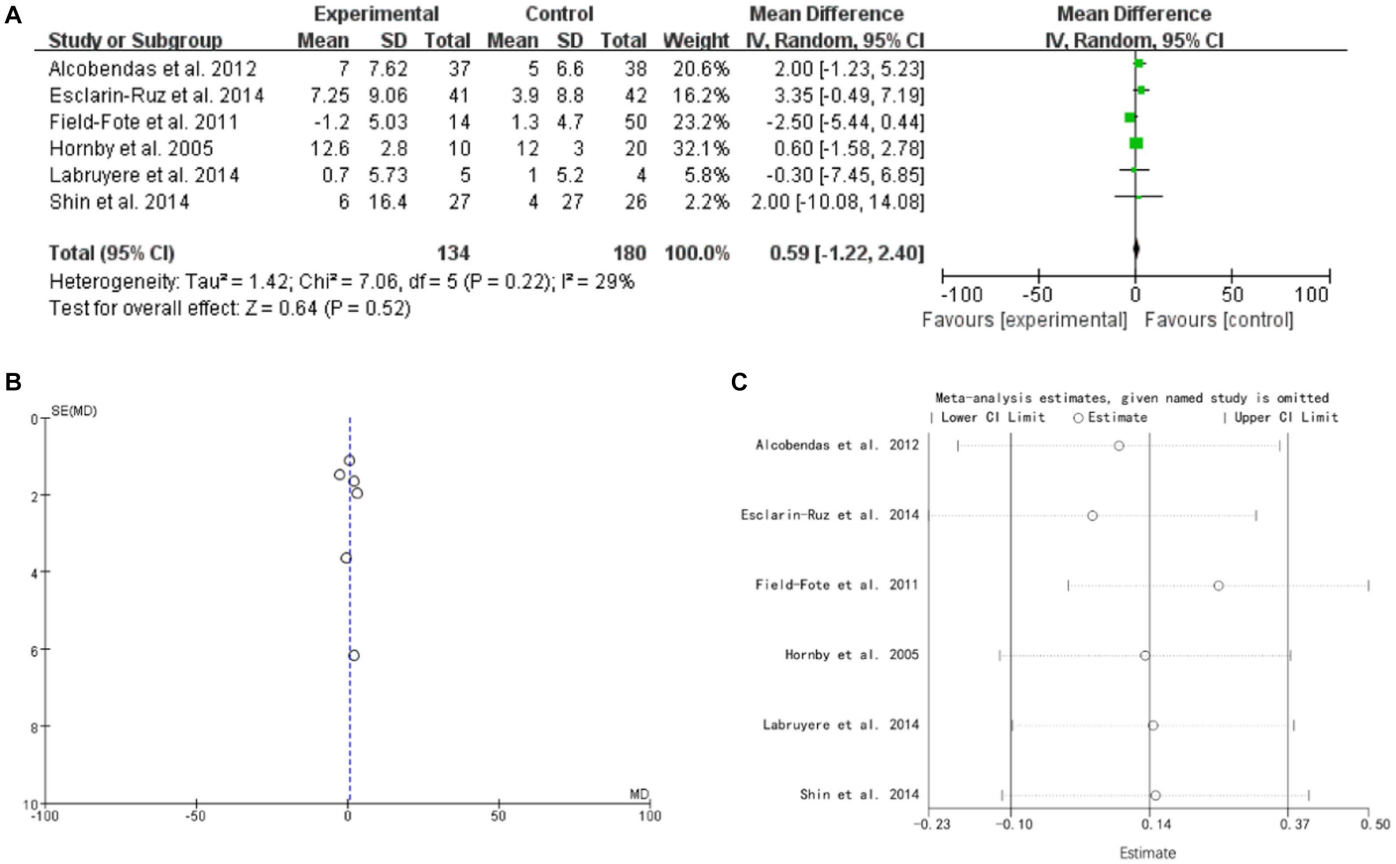
Leg strength **(A)** forest plot, **(B)** funnel plot, and **(C)** sensitivity analysis.

6 studies mentioned leg strength. The forest plot weight mean difference WMD = 0.59, 95% CI (−1.22, 2.40), I^2^ = 29%. The funnel plot shows that asymmetric. It may have publication bias. Sensitivity analysis is conducted and show that values included in the literature are all within a reasonable range (Figure 5).

### 3.7. Timed up and go

**Figure 6:**
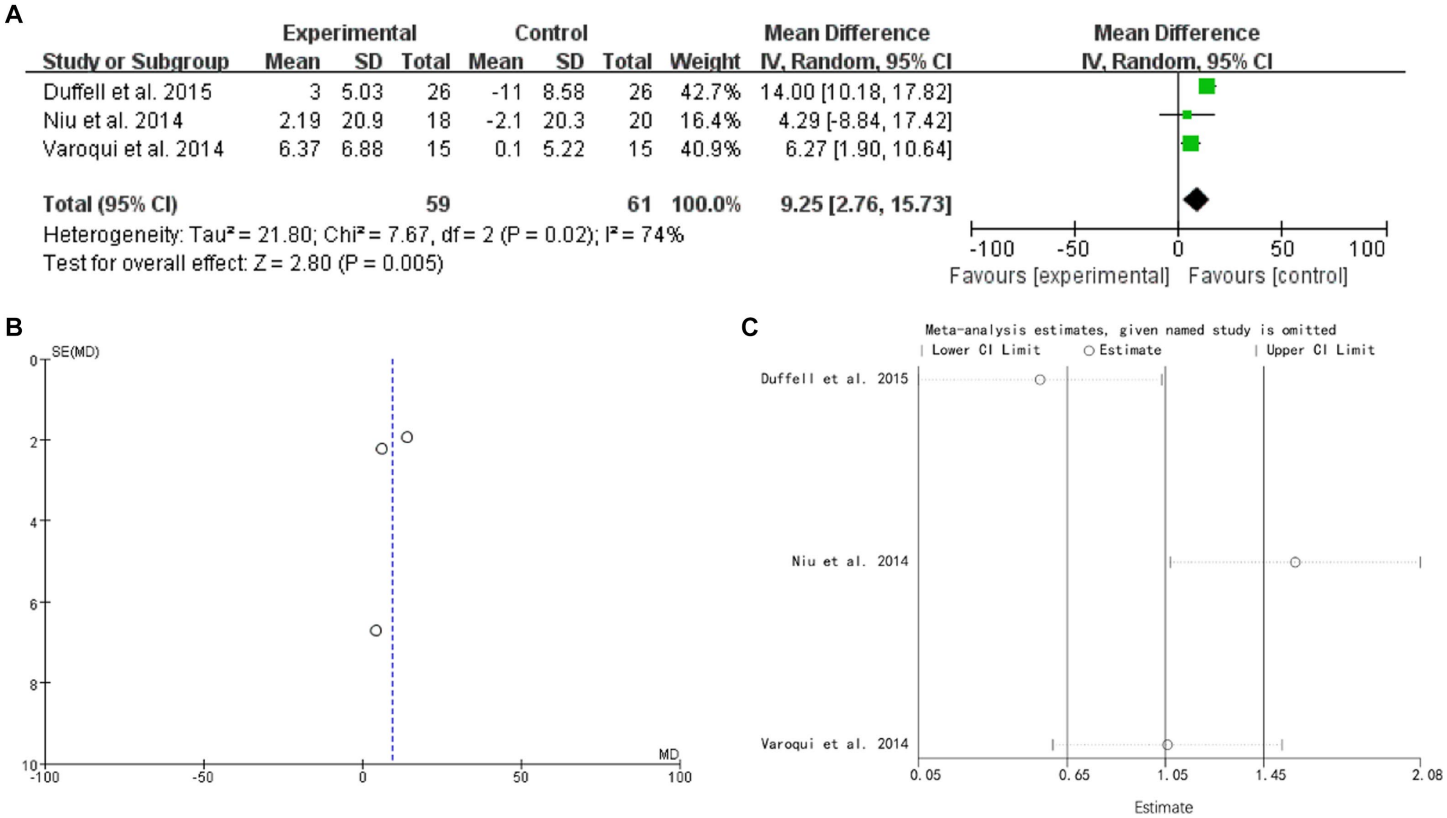
TUG **(A)** forest plot, **(B)** funnel plot, and **(C)** sensitivity analysis.

3 studies mentioned TUG. The forest plot weight mean difference WMD = 9.25, 95% CI (2.76, 15.73), I^2^ = 74%. The funnel plot shows that asymmetric. It may have publication bias. Sensitivity analysis is conducted and show that values included in the literature are all within a reasonable range (Figure 6).

### 3.8. 10 MWT

**Figure 7:**
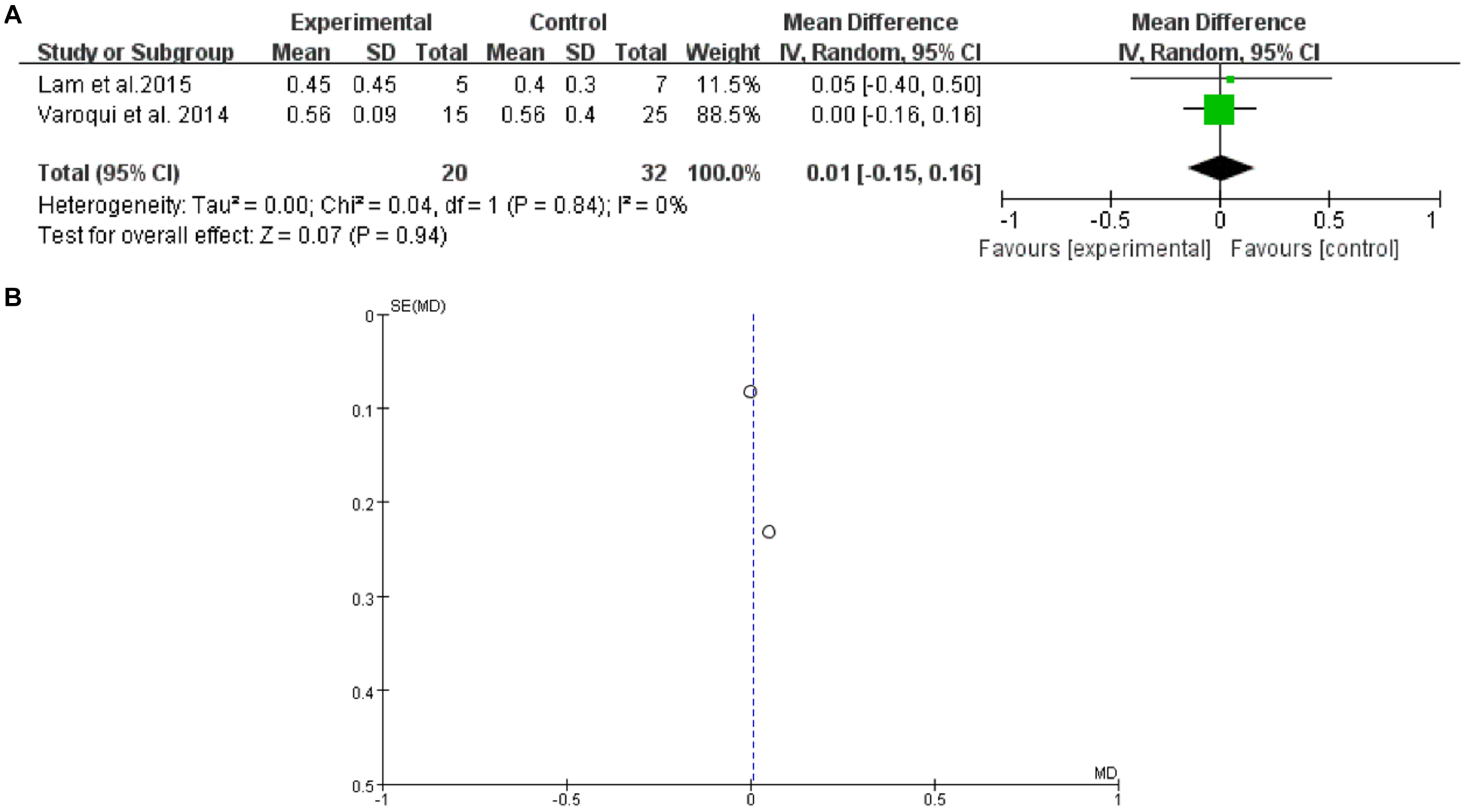
10 MWT **(A)** forest plot and **(B)** funnel plot.

2 studies mentioned 10 MWT. The forest plot weight mean difference WMD = 0.01, 95% CI (−0.15, 0.16), I^2^ = 0%. The funnel plot shows that asymmetric. It may have publication bias (Figure 7).

### 3.9. 6 MWT

**Figure 8:**
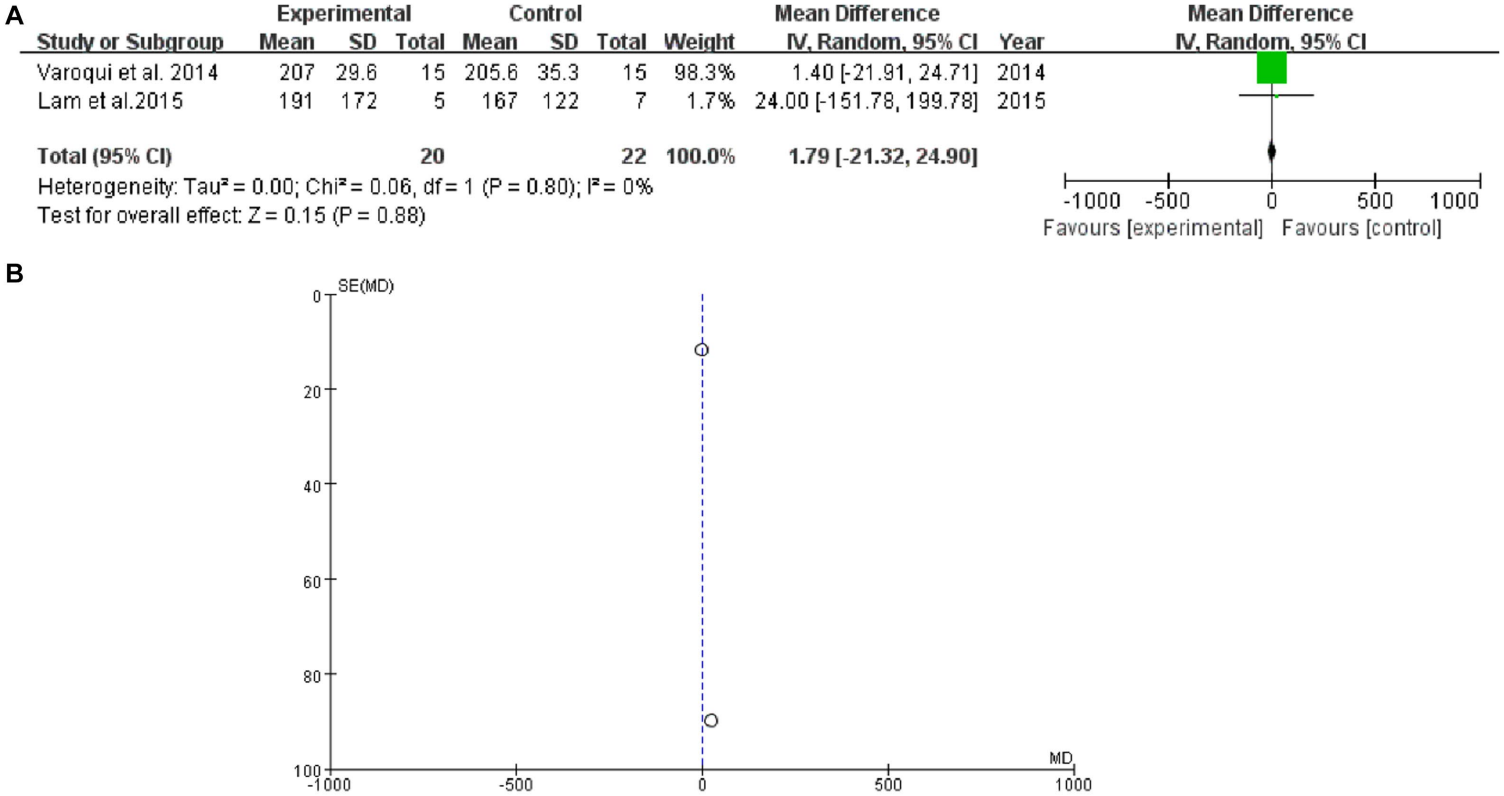
6 MWT **(A)** forest plot and **(B)** funnel plot.

2 studies mentioned 6 MWT. The forest plot weight mean difference WMD = 1.79, 95% CI (−21.32, 24.90), I^2^ = 0%. The funnel plot shows that asymmetric. It may have publication bias (Figure 8).

### 3.10. Safety

No studies have reported the occurrence of adverse events.

## 4. Discussion

1,520 records were found by searching the database, and 11 studies were finally selected for the meta-analysis. All studies included were deemed to have low risk of selection bias. However, due to the use of various rehabilitation methods, physicians inevitably know the differences in intervention methods used. All studies were found to have a high risk of performance bias and detection bias. Additionally, all studies were found to have a low risk of attrition bias and reporting bias. However, some studies had unclear risks of other biases, such as lost follow-up. The result showed that gait distance [WMD = 16.05, 95% CI (−15.73, 47.83), I2 = 69%], gait speed (RAGT vs. regular treatment) [WMD = 0.01, 95% CI (−0.04, 0.05), I2 = 43%], gait speed (RAGT vs. no intervention) [WMD = 0.07, 95% CI (0.01, 0.12), I2 = 0%], leg strength [WMD = 0.59, 95% CI (−1.22, 2.40), I2 = 29%], TUG [WMD = 9.25, 95% CI (2.76, 15.73), I2 = 74%], 10 MWT [WMD = 0.01, 95% CI (−0.15, 0.16), I2 = 0%], and 6 MWT [WMD = 1.79, 95% CI (−21.32, 24.90), I2 = 0%]. Robot-assisted gait training appears to be beneficial in enhancing TUG for patients with spinal cord injuries. However, it may not have an impact on gait distance, gait speed, leg strength, 10 MWT, and 6 MWT. The quality of the evidence level is not high because the included articles have high bias of risks.

According to the current results, the lack of significant aid from machine-assisted rehabilitation can be attributed to several factors (Bowman et al., 2021) Firstly, the current technology for machine-assisted rehabilitation is not advanced enough to fully replace human rehabilitation (Hayes et al., 2018). The equipment’s functions and applicability are limited, making it difficult to cater to the rehabilitation needs of all patients. Secondly, each person’s physical state and rehabilitation requirements are unique, and machine-assisted rehabilitation equipment cannot provide customized plans tailored to individual circumstances (Aguirre-Güemez et al., 2019). Thirdly, professional medical personnel are required to provide guidance and supervision for machine-assisted rehabilitation, and a lack of professional guidance can result in inadequate outcomes (Haji Hassani et al., 2022). Finally, long-term monitoring and evaluation are necessary for the rehabilitation effect of machine-assisted rehabilitation equipment, and the absence of such monitoring can lead to less significant rehabilitation outcomes (Alen et al., 2014).

Although in current research, the effectiveness of robot assisted rehabilitation therapy is not significant. There is still room for research in its development. With the continuous development of technology, the application of robots in the medical field is becoming increasingly extensive. In rehabilitation therapy, robots can provide accurate and precise motion control to assist patients in their recovery training. Robot-assisted rehabilitation therapy has advantages such as providing personalized treatment, enhancing patient engagement, improving treatment outcomes, and reducing the burden on healthcare professionals, making it an important trend in the field of rehabilitation therapy.

Our research has several strengths. While previous Cochrane Reviews have shown that the effect of spinal cord injury, they did not analyze the impact on spinal cord injury (Boldt et al., 2014). Additionally, other meta-analyses have been limited by their use of English-language studies only. In contrast, our systematic review includes studies in Chinese, making it a more comprehensive and up-to-date analysis of the role of robot-assisted gait training for patients with spinal cord injury. However, our study also has some limitations. Firstly, most of the studies we included did not use blinding methods and had a high risk of bias. Secondly, we were unable to assess the specific type of usually PT used in each study due to a lack of randomized controlled trials.

This study has important implications for future research in the field of clinical rehabilitation. To further advance our understanding of the benefits of Tai Chi, it is recommended that future studies include longer-term follow-up periods, as well as more randomized controlled trials and mechanism research. Additionally, many of the studies included in our analysis either inadequately reported or did not clearly report important methodological details such as randomization/allocation concealment and blinding methods. To improve the quality of reporting in future trials, we recommend that researchers adhere to the Consolidated Standards of Reporting Trials (CONSORT) statement (Schulz et al., 2010).

## Data availability statement

The raw data supporting the conclusions of this article will be made available by the authors, without undue reservation.

## Author contributions

LB, XW, and FD collected data. WQ, MiY, and SY performed the analysis and interpretation of data. LB, HJ, and XW wrote the manuscript. MaY designed the study. All authors contributed to the article and approved the submitted version.

## Funding

This work was supported by Changshu Science and Technology Bureau Project (CS202001) and Changshu Traditional Chinese Medicine Hospital Youth Project (cszyy201914).

## Conflict of interest

The authors declare that the research was conducted in the absence of any commercial or financial relationships that could be construed as a potential conflict of interest.

## Publisher’s note

All claims expressed in this article are solely those of the authors and do not necessarily represent those of their affiliated organizations, or those of the publisher, the editors and the reviewers. Any product that may be evaluated in this article, or claim that may be made by its manufacturer, is not guaranteed or endorsed by the publisher.

## Abbreviations

6MWT: 6-min walk test
10MWT: 10-meter walk test
PT: physical therapy
RAGT: robot-assisted gait training
RCTs: randomized controlled trials
TUG: timed up and go
